# Postoperative Bleb Management with Topical Mitomycin-C

**Published:** 2011-04

**Authors:** Jost B Jonas

**Affiliations:** Department of Ophthalmology, Mannheim Medical Faculty of the Ruprecht-Karls-University, Heidelberg, Germany

Despite various developments in many fields of ophthalmology, the surgical therapy of open-angle glaucoma has remained mostly unchanged over the last 30 years. Ever since the introduction of intraoperative mitomycin and postoperative subconjunctival injections of 5-fluorouracil (5-FU), trabeculectomy has not markedly been altered. These facts stand in contrast to the need for a more efficient surgical therapy for open-angle glaucoma, since one of the main disadvantages of filtering surgery is the lack of enduring success. Large-scale retrospective studies have suggested that filtering blebs become non-functional in at least 50% of patients, 5 years after surgery. This clearly demonstrates the necessity for developing techniques to reduce postoperative scarring in filtering blebs.

In the current issue of Journal of Ophthalmic and Vision Research, Pakravan and colleagues^1^ have examined the effect of postoperative topical mitomycin 0.02% eye drops on the success of trabeculectomy surgery and compared the results with that of repeated postoperative subconjunctival 5-FU injections. In their randomized study, for which the authors have to be commended, the authors found that topical mitomycin compared favorably with subconjunctival 5-FU injections. Since eye drops are easier to apply and cause less pain, topical application of mitomycin appears to be a valid alternative to subconjunctival 5-FU injections in the postoperative period.

Certain questions however remain. First, interestingly, the toxicity of mitomycin applied topically on intact conjunctival epithelium is considerably less than that applied on bare sclera. Future studies may address whether this disparity is due to different durations of application (drops have relatively short period of contact with the ocular surface), variations in effective concentrations of the agent (drops may be diluted by tears), and/or due to the protective effect of an intact conjunctival epithelium. Second, it is not clear yet whether prolonged topical application of mitomycin may cause corneal stem cell insufficiency leading to problems in corneal epithelialization or dry eye due to damage to conjunctival goblet cells. Third, the risk of systemic side effects of mitomycin eye drops due to absorption through the nasolacrimal duct mucosa may additionally be addressed.

In conclusion, the study by Pakravan et al may show new possibilities and open up a new avenue in the postoperative care of patients after routine trabeculectomy. Since the application of mitomycin eye drops is considerably more convenient and probably less costly, it may indeed be an alternative to postoperative subconjunctival injections of 5-FU.

## References

[1] Pakravan M, Miraftabi A, Yazdani S, Koohestani N, Yaseri M (2011). Mitomycin-C drops versus subconjunctival 5-fluorouracil for management of bleb failure. J Ophthalmic Vis Res.

